# Case Report: Prophylactic Plate Fixation for Incomplete Atypical Ulnar Fractures Resulting From the Use of Denosumab for Bone Metastases

**DOI:** 10.3389/fendo.2021.798653

**Published:** 2022-01-07

**Authors:** Atsuro Murai, Kaoru Tada, Tadahiro Nakajima, Mika Akahane, Masashi Matsuta, Yuta Nakamura, Hiroyuki Tsuchiya

**Affiliations:** Department of Orthopaedic Surgery, Graduate School of Medical Science Kanazawa University, Kanazawa, Japan

**Keywords:** atypical fracture, bone metastases, prophylactic fixation, ulna, denosumab, severely suppression of bone turnover

## Abstract

Patients with bone metastases are treated with long-term bone resorption inhibitors such as bisphosphonates and denosumab. However, resorption inhibitors have been known to cause fractures, such as atypical femoral fractures (AFFs). In recent years, there have been an increasing number of reports of atypical ulna fractures (AUFs) caused by bone resorption inhibitor usage. Treatment of AUFs is complicated, especially when they occur in patients with bone metastases, because it is difficult to discontinue bone resorption inhibitor treatment without the risk of aggravating metastatic lesions. Prophylactic surgery is recommended in AFFs when fractures are predicted, but there are few reports of prophylactic surgery for AUFs. Here, we report a case of incomplete AUF in a 74-year-old woman which was surgically treated with prophylactic plate fixation. The patient had been using denosumab for 6 years to treat bone metastases due to thyroid cancer. After surgery, no fractures were observed for 2 years without discontinuing denosumab, and her forearm function was adequate. AUFs are rare and difficult to treat, so oncologists who treat bone metastases need to pay special attention to diagnose this incomplete AUF before the fracture worsens. We believe that detection of a possible fracture and prophylactic surgery can improve prognosis.

## Introduction

The use of bone resorption inhibitors such as bisphosphonates and denosumab is recommended for the treatment of patients with bone metastases, and is administered long-term to prevent skeletal-related events (1, 2). However, bone resorption inhibitors can cause severe suppression of bone turnover (SSBT). In 0.9-9.7% of patients with bone metastases, the use of bone resorption inhibitors has been reported to cause atypical femoral fractures (AFFs) (3–6). Furthermore, in recent years, there have been increasing incidence of atypical ulnar fractures (AUFs) in addition to AFFs caused by SSBT (7, 8). AUF treatment is difficult, and many authors have reported that bone union is not achieved (9, 10). Therefore, the discontinuation of bone resorption inhibitors, as well as the use of bone grafting, dual plate fixation, low-intensity pulsed ultrasound (LIPUS), and teriparatide are recommended (9, 11, 12). However, discontinuation of bone-resorbing drugs is problematic in patients with bone metastases, and AUFs treatment in such patients has been reported to be challenging (11). Prophylactic intramedullary nail fixation is recommended for AFFs when an incomplete fracture occurs because of the treatment (13). However, there are few reports of prophylactic fixation of AUFs, especially in patients using bone resorption inhibitors for bone metastasis treatment. Here, we report a case of prophylactic plate fixation of an incomplete AUF in a patient who had been on denosumab for 6 years. She had no fractures for 2 years after surgery while continuing treatment with bone resorption inhibitors.

## Case Presentation

**Figure 1:**
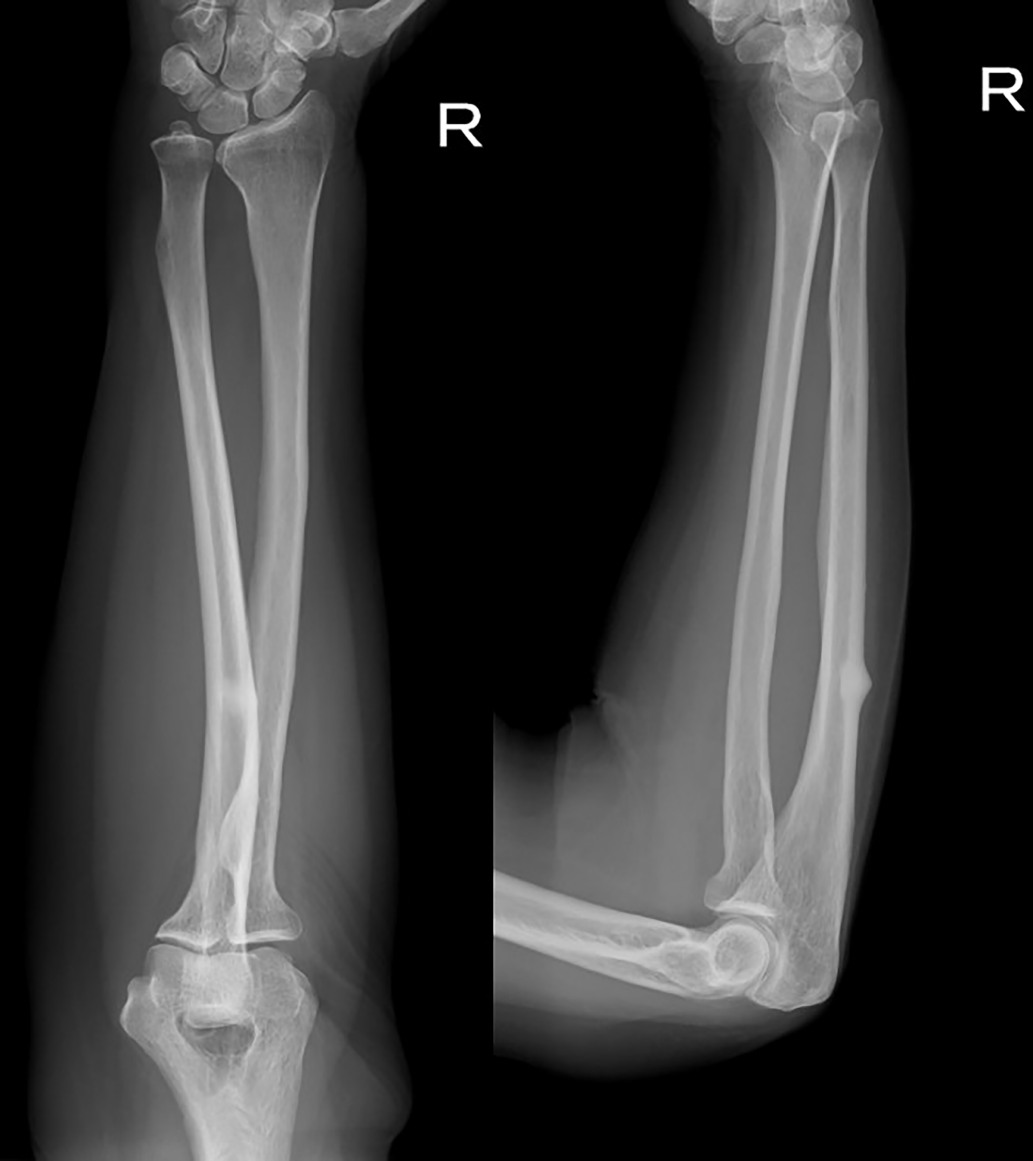
Radiograph at time of diagnosis. Periosteal and endosteal thickening of the dorsal cortex and sclerotic changes in the proximal third of the ulna.

A 74-year-old woman experienced a protrusion with associated pain in her right forearm. Her medical history included left thyroid lobectomy at the age of 43 for thyroid follicular carcinoma. At 53 and 56 years, metastatic tumors were detected in her left sixth rib and the first lumbar vertebra, and resection for the left sixth rib and total en bloc spondylectomy for the first lumbar vertebra were performed. However, at the age of 58 years, bone metastases were detected in the left iliac crest and radiation therapy, bisphosphonates (incadronate) intravenous administration (10 mg every 2 weeks) and calcitriol oral administration (0.5 µg per day) were initiated. At the age of 65 years, she underwent tumor resection for metastasis in the eleventh thoracic vertebra, and as the supply of incadronate injection was discontinued in our country, the patient began treatment with zoledronic acid (3 mg per month); however, due to worsening renal dysfunction, the medication was switched to denosumab (120 mg per month) when the patient was 68 years old. She had a right AFF at the age of 72 years and underwent intramedullary nail fixation. In the current report, physical examination revealed mild protrusion and tenderness in the proximal ulna. Radiography revealed periosteal and endosteal thickening of the dorsal cortex with sclerotic changes in the proximal third of the ulna (**Figure 1**). Blood test results showed normal serum bone metabolic marker levels; moreover, ^18^F-fluorodeoxyglucose-positron emission tomography did not reveal any abnormal uptake in the proximal third of the ulna. These findings led to the diagnosis of osteosclerosis with cortical thickening as a result of incomplete AUF. We intended to discontinue denosumab but were unable to do so, as it was required for the treatment of her bone metastases. Additionally, the patient used a walking frame for ambulation and continued to stress her upper extremities. Therefore, the ulna was at a high risk of a complete fracture and prophylactic osteosynthesis was planned.

**Figure 2:**
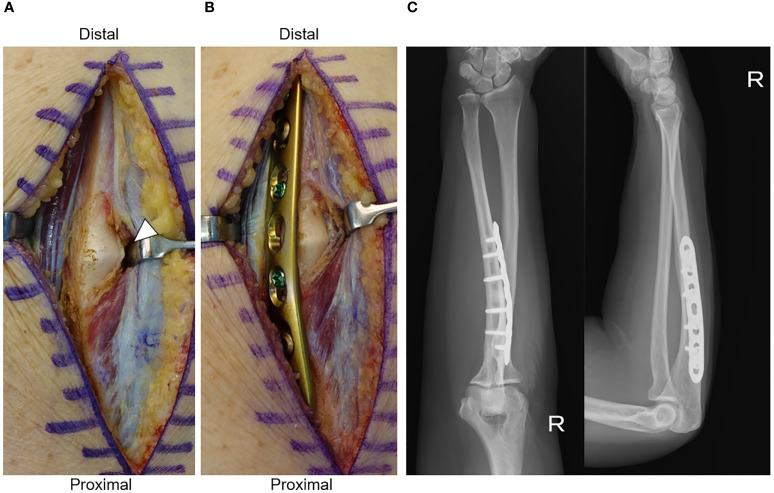
**(A)** Operative findings before plate fixation. White arrow head shows thickening of the ulnar cortex. **(B)** Operative findings after plate fixation. **(C)** Radiograph 2 years after surgery.

The surgery was performed under general anesthesia with the patient in the supine position. Cortical thickening was observed between the extensor carpi ulnaris and flexor carpi ulnaris (**Figure 2A**). The locking plate (LC-LCP3.5; DePuy Synthes, Zeist, Netherlands) was bent to conform to the bone shape and fixed in position (**Figure 2B**).

Postoperatively, the forearm pain disappeared. Furthermore, 2 years postoperatively, there was no limitation in the range of motion of the right upper extremity, no significant increase in cortical bone thickening was observed, and no fracture had occurred (**Figure 2C**).

## Discussion

Reports of AUFs have increased in recent years, and is believed to be more common than previously reported (7).

Although there are no clear diagnostic criteria for AUFs, they are generally suspected when the following characteristics are present: old age, female sex, sustained atraumatic or minor trauma, discomfort in the proximal third of the ulna, noncomminuted transverse or short oblique fractures, complete or incomplete fractures, and cortical thickening and sclerosis at the fracture site (8, 14).

Bisphosphonates are the most commonly reported drugs causing AUFs (7, 11), while denosumab has also been reported to cause AUF (15). In this case, the patient had been treated with incadronate, zoledronic acid, and denosumab. The risk of atypical fractures has been reported to decrease rapidly after discontinuation of bisphosphonates (16), and she discontinued treatment with incadronate 9 years ago and zoledronic acid 6 years ago. Therefore, we diagnosed an incomplete AUF with denosumab as the causative agent.

Unlike patients who use bone resorption inhibitors only for osteoporosis, patients with bone metastases cannot discontinue bone resorption inhibitors or use teriparatide. Pseudoarthrosis and plate fracture have been reported in patients with bone metastases who underwent plate fixation, vascularized bone grafting, and even LIPUS for AUF (11). This indicates that treatment of AUFs in patients with bone182 metastases is very challenging. Therefore, prophylactic fixation, before a complete fracture occurs, may be a useful treatment for patients with bone metastases.

With the development of cancer treatment, the life expectancy of patients with bone metastases is increasing, and the treatment of bone metastases is likely to be prolonged. Long-term administration of bone resorption inhibitors is associated with the well-known risks of osteonecrosis of the jaw and AFF (2). In addition, oncologists treating bone metastases also need to be aware of AUF because of the difficulty in treating this rare type of fracture.

Unlike AFFs, the upper extremities are not weight-bearing sites, and it has been reported that patients are less likely to report pain before a complete fracture occurs (10), making it difficult to detect incomplete AUFs. In this case, we were fortunate to be able to detect a fracture because the patient complained of pain, probably due to the use of a walking frame and weight-bearing upper extremities.

Routine radiographic screening for AFFs is recommended for patients receiving long-term bone resorption inhibitors for the treatment of bone metastases (1). We also recommend routine radiographic screening of AUFs. Our report suggests that if an incomplete AUF is observed, prophylactic plate fixation may prevent fractures while continuing treatment with bone resorption inhibitors. Early detection of incomplete AUFs and prophylactic plate fixation may improve the patient’sprognosis.

## Data Availability Statement

The original contributions presented in the study are included in the article/supplementary material. Further inquiries can be directed to the corresponding author.

## Ethics Statement

Written informed consent was obtained from the individual(s) for the publication of any potentially identifiable images or data included in this article.

## Author Contributions

KT, TN, and MA performed surgery and AM, KT, MM, and YN were monitoring the patient’s post-operative progress. AM drafted the first manuscript. KT, TN, MA, MM, YN, and HT provided supervision and participated in the literature review and in drafting the manuscript. All authors read and approved the final manuscript.

## Conflict of Interest

The authors declare that the research was conducted in the absence of any commercial or financial relationships that could be construed as a potential conflict of interest.

## Publisher’s Note

All claims expressed in this article are solely those of the authors and do not necessarily represent those of their affiliated organizations, or those of the publisher, the editors and the reviewers. Any product that may be evaluated in this article, or claim that may be made by its manufacturer, is not guaranteed or endorsed by the publisher.
